# Inflammatory Biomarkers and Platelet Indices in Odontogenic and Nonodontogenic Head and Neck Abscesses: A Prospective Observational Study

**DOI:** 10.7759/cureus.114712

**Published:** 2026-08-18

**Authors:** Gergana М Chausheva, Yanko G Yankov, Diana D Nenova

**Affiliations:** 1 Department of Clinical Laboratory, Medical University "Prof. Dr. Paraskev Stoyanov", Varna, BGR; 2 Department of Central Clinical Laboratory, University Hospital "St. Marina", Varna, BGR; 3 Clinic of Maxillofacial Surgery, University Hospital "St. Marina", Varna, BGR; 4 Department of General and Operative Surgery, Medical University "Prof. Dr. Paraskev Stoyanov", Varna, BGR; 5 Department of Nephrology, Medical University "Prof. Dr. Paraskev Stoyanov", Varna, BGR; 6 Clinic of Nephrology and Dialysis, University Hospital "St. Marina", Varna, BGR

**Keywords:** c-reactive protein, head and neck surgery, inflammation, maxillofacial surgery, mean platelet volume, nonodontogenic abscess, odontogenic abscess, oral surgery, platelet indices, procalcitonin

## Abstract

Introduction: Odontogenic and nonodontogenic head and neck abscesses differ in their etiology and clinical presentation. However, data comparing inflammatory biomarkers and platelet (PLT) indices in these conditions remain limited. The aim was to compare serum procalcitonin (PCT), C-reactive protein (CRP), white blood cell count (WBC), PLT, mean platelet volume (MPV), and the MPV-to-PLT ratio (MPR) between patients with odontogenic and nonodontogenic abscesses and to assess their associations with abscess origin and with each other.

Methods: This prospective observational study included 80 adult patients with head and neck abscesses (50 odontogenic and 30 nonodontogenic), hospitalized at the Clinic of Maxillofacial Surgery, University Hospital "St. Marina," Varna, Bulgaria, from the beginning of July 2021 to the end of June 2022. PLT indices (PLT and MPV) and WBC were obtained from a complete blood count analysis using an automated 5-part differential hematology analyzer (ADVIA 2020, Siemens Healthineers, Erlangen, Germany). CRP and PCT levels were quantified using immunoturbidimetric analysis (CRP on Cobas® 6000, Roche Diagnostics, Mannheim, Germany; PCT on ADVIA 1800, Siemens Healthineers). Statistical analyses were performed using the Statistical Package for the Social Sciences, version 19 (IBM Corp., Armonk, NY).

Results: CRP concentrations were significantly higher in patients with odontogenic abscesses than in those with nonodontogenic abscesses (68.79 (29.46-143.08) vs. 12.29 (3.46-41.32) mg/L, p < 0.001; rᵣᵦ = 0.55, 95% confidence interval (CI): 0.31-0.75). In contrast, no significant between-group differences were observed in PCT, WBC, PLT, MPV, or MPR (all p > 0.05). In multivariable analysis, higher PCT concentrations were independently associated with nonodontogenic abscess origin (adjusted odds ratio (OR) = 2.06, 95% CI: 1.23-3.45, p = 0.006), whereas higher CRP concentrations were associated with lower odds of nonodontogenic origin (adjusted OR = 0.981, 95% CI: 0.969-0.992, p = 0.001). Significant positive correlations between CRP and WBC were observed in both the odontogenic (ρ = 0.585, p < 0.001) and nonodontogenic (ρ = 0.546, p = 0.002) groups. In the nonodontogenic group, MPV was negatively correlated with PLT (ρ = -0.466, p = 0.010), while PLT was positively correlated with WBC (ρ = 0.442, p = 0.014).

Discussion: The findings indicate different patterns of association of CRP and PCT with abscess origin. Although only CRP differed significantly between the groups in the unadjusted analysis, multivariable analysis identified independent associations of both CRP and PCT with abscess origin. MPV and MPR appear to have limited value in distinguishing odontogenic from nonodontogenic abscesses.

Conclusion: CRP and PCT showed different patterns in the two types of head and neck abscesses. Although only CRP differed significantly between the groups in the unadjusted analysis, higher PCT was independently associated with nonodontogenic abscess origin, whereas higher CRP was associated with lower odds of nonodontogenic origin in multivariable analysis. PLT indices, including PLT, MPV, and MPR, showed no significant between-group differences.

## Introduction

One of the most common reasons for emergency hospitalization of patients in maxillofacial surgery clinics is purulent infections of the soft tissues of the head and neck, called abscesses and phlegmons. They are caused by pathogenic microorganisms or by the patient's own resident microflora and often affect young individuals [1,2]. Depending on their etiology, they are divided into odontogenic and nonodontogenic abscesses and phlegmons [3]. If left untreated, the infection can enter the bloodstream and lymph and spread to other regions, causing serious systemic reactions [4]. In odontogenic infections, the etiological agent enters the jawbones through damaged dental or adjacent tissues and subsequently spreads into the surrounding soft-tissue spaces, resulting in purulent inflammation [5]. The causes of nonodontogenic infections of the head and neck can be very different, but the most common are inflamed skin cysts and impaired integrity of the skin or mucosal surface, where pathogenic microorganisms enter and cause purulent inflammation of the adjacent soft tissues [6]. Although the two types of purulent infection differ in their mechanisms of occurrence, their management is generally similar and includes surgical drainage of purulent exudate combined with oral or intravenous antibacterial therapy [7]. However, they differ in their clinical manifestation and degree of influence on the inflammatory indicators used in their diagnosis and treatment [5-7].

Although numerous studies have investigated odontogenic and nonodontogenic head and neck infections, limited evidence is available regarding differences in procalcitonin (PCT), C-reactive protein (CRP), white blood cell (WBC) count, and platelet (PLT) indices (mean platelet volume (MPV), PLT, and the MPV-to-PLT ratio (MPR)) according to abscess origin. Therefore, the aim of the present study was to compare inflammatory biomarkers and PLT indices between patients with odontogenic and nonodontogenic head and neck abscesses and to investigate their associations with abscess origin and with each other.

## Materials and methods

This prospective observational study was conducted over a period of 12 months at the Clinic of Maxillofacial Surgery, University Hospital "St. Marina," Varna, Bulgaria, from the beginning of July 2021 to the end of June 2022. The study included 80 patients admitted for emergency surgical treatment of head and neck abscesses who met the predefined inclusion and exclusion criteria.

The present study represents a secondary analysis of a previously established prospective cohort conducted under the same Institutional Review Board approval (Approval No. 101/24.03.2021), as both investigations originated from the same prospective observational study but addressed different research questions.

The inclusion criteria were that the patients were 18 years of age or older, were hospitalized, had a purulent exudate on the head and/or neck that was evacuated during the surgical treatment, and had preserved medical documentation allowing the categorical distinction between odontogenic and nonodontogenic etiology of the infection, and had signed an informed consent to participate in the study.

Exclusion criteria were patients under 18 years of age, those who did not agree to participate in the study, patients in whom there was no definite data on the etiology of the infection (odontogenic or nonodontogenic), who were not hospitalized, and those who had some of the factors and conditions known to affect the investigated laboratory parameters ​​of the studied and analyzed indicators in the present study.

During the study period, 81 consecutive patients with purulent head and neck infections were admitted to the Clinic of Maxillofacial Surgery for emergency surgical treatment. One patient was excluded because of a concomitant lymphoproliferative disorder that did not meet the predefined eligibility criteria. Consequently, 80 patients were included in the final analysis and were classified according to the origin of the infection into two groups: 50 patients with odontogenic abscesses and 30 with nonodontogenic abscesses. In all cases, the diagnosis of a head and neck abscess was established by an oral and maxillofacial surgeon based on the clinical examination and was confirmed intraoperatively by the presence of purulent exudate during surgical drainage. Odontogenic origin was determined based on clinical history and examination, including a history of toothache or the presence of decayed teeth, and, when available, imaging evidence of dental pathology considered to be associated with the infection. Abscesses without evidence of odontogenic origin were classified as nonodontogenic. CT imaging was not mandatory for classification, and the assessment of abscess origin was not performed in a blinded manner. No a priori sample size calculation was performed. All eligible patients admitted during the 12-month study period were consecutively included, and the final sample size was determined by the number of patients meeting the inclusion and exclusion criteria.

All participants underwent a uniform clinical and laboratory assessment at enrollment. Demographic and clinical information was retrieved from the corresponding medical records. Venous blood samples were collected prior to surgical drainage, in the morning after an overnight fast of at least 12 hours, and were processed within two hours of collection. Serum was isolated by centrifugation of blood collected in gel-separator tubes at 2,500 × g for 15 minutes. For complete blood count analyses, whole blood was collected into tubes containing dipotassium ethylenediaminetetraacetic acid as the anticoagulant.

Complete blood count analysis, including WBC, PLT count, and MPV, was performed with an ADVIA 2020 automated 5-part differential hematology analyzer (Siemens Healthineers, Erlangen, Germany) following the manufacturer's recommendations. Cell differentiation is based on flow cytometry using light scatter, differential leukocyte lysis, myeloperoxidase activity, and oxazine 750 staining. PLT was obtained by the impedance method, while MPV was generated automatically by the instrument's integrated analytical software. To reduce the effect of ethylenediaminetetraacetic-induced PLT swelling, MPV was determined within two hours after blood collection. Laboratory results were expressed as follows: WBC and PLT in ×10⁹/L, MPV in femtoliter, while the MPV-to-platelet count ratio (MPR) was calculated as the quotient of MPV and PLT and expressed as a numerical value. Serum samples were used for the determination of CRP and PCT. CRP concentrations were measured by a latex-enhanced immunoturbidimetric assay on the Cobas® 6000 analyzer (Roche Diagnostics, Mannheim, Germany). Serum PCT concentrations were determined using a latex-enhanced immunoturbidimetric method on the ADVIA 1800 clinical chemistry analyzer (Siemens Healthineers, Erlangen, Germany) with Diazyme reagents (Diazyme Laboratories Inc., Poway, CA). CRP and PCT results were reported in milligram/liter and nanogram/milliliter, respectively.

Statistical analysis was carried out using IBM Statistical Package for the Social Sciences Statistics, version 19 (IBM Corp., Armonk, NY) running on the Windows 10 operating system (Microsoft Corp., Redmond, WA). Continuous variables were summarized according to their distribution, while categorical variables were presented as frequencies and percentages. The distribution of continuous variables was assessed using the Shapiro-Wilk test, together with skewness and kurtosis. Histograms and normal probability plots were additionally inspected. Normally distributed variables were presented as mean ± standard deviation, whereas variables with nonnormal distribution were expressed as median and interquartile range. Given the exploratory nature of the study, no formal adjustment for multiple comparisons was applied; therefore, secondary and subgroup analyses should be interpreted cautiously.

Differences between two independent groups were assessed using the independent-samples Student's t-test for normally distributed variables and the Mann-Whitney U test for variables with nonnormal distribution. Cohen’s d was used as the effect size measure for significant parametric comparisons, whereas rank-biserial correlation (rᵣᵦ) was used for significant nonparametric comparisons, with corresponding 95% confidence intervals (CIs). Categorical variables were compared using Pearson's chi-square test. Pearson's or Spearman's correlation coefficients were used for normally and nonnormally distributed continuous variables, respectively. A multivariable binary logistic regression analysis was performed to evaluate the independent associations of CRP and PCT with abscess origin. Abscess origin (nonodontogenic = 1, odontogenic = 0) was entered as the dependent variable, while CRP, PCT, age, and sex were included as independent variables. Adjusted odds ratios (ORs) with 95% CIs were reported. All statistical analyses were two-tailed, and statistical significance was defined as a p value of <0.05.

## Results

Gender distribution was comparable between the two groups (Pearson's χ² test, χ²=0.889, p = 0.346). The mean age was 41.96 ± 18.18 years in the odontogenic abscess group and 44.53 ± 15.49 years in the nonodontogenic abscess group, with no significant difference between the groups (independent-samples Student's t-test, t = -0.65, p = 0.52). A summary of the baseline characteristics is presented in Table 1.

**Table 1:** Baseline characteristics Data are presented as median (IQR). Between-group comparisons were performed using the Mann-Whitney U test ^*^Statistical significance, defined as a two-sided p value of <0.05 IQR: interquartile range; WBC: white blood cell; MPV: mean platelet volume; PLT: platelet; MPR: mean platelet volume-to-platelet count ratio; CRP: C-reactive protein; PCT: procalcitonin

Parameter	Patients with odontogenic abscesses (n = 50), median (IQR)	Patients with nonodontogenic abscesses (n = 30), median (IQR)	Test statistic (U)	Reference ranges	p value
WBC, ×10^9^/L	10.35 (7.76-12.76)	9.16 (7.89-12.78)	799.5	3.79-10	0.626
MPV, fL	8.30 (7.70-9.30)	8.35 (7.60-9.18)	796.5	6.0-10	0.647
PLT, ×10^9^/L	265.0 (216.0-326.25)	279.5 (207.75-333.75)	712.5	140-440	0.713
MPR, MPV/PLT	0.0313 (0.0268-0.0379)	0.0310 (0.0248-0.0429)	793.0	-	0.673
CRP, mg/L	68.79 (29.46-143.08)	12.29 (3.46-41.32)	1,159.0	0-5	<0.001^*^
PCT, ng/mL	0.375 (0.273-0.725)	0.405 (0.253-2.535)	675.0	0-0.05	0.459

CRP concentrations were significantly higher in patients with odontogenic abscesses than in those with nonodontogenic abscesses (68.79 (29.46-143.08) vs. 12.29 (3.46-41.32) mg/L; Mann-Whitney U = 1,159.0, p < 0.001), with a large effect size (rank-biserial correlation, rᵣᵦ = 0.55; 95% CI: 0.31-0.75). Among patients with nonodontogenic abscesses, CRP concentrations were numerically higher in men than in women (23.31 (3.79-56.48) vs. 9.73 (1.95-15.42) mg/L), although the difference did not reach statistical significance (Mann-Whitney U = 139.0, p = 0.090). Women had higher PLT than men (294 (281-348) vs. 234 (202.75-300.75) × 10⁹/L), but this difference was also not statistically significant (U = 71.5, p = 0.218). No significant sex-related difference was observed in MPR (0.0275 (0.0248-0.0307) in women vs. 0.0360 (0.0255-0.0491) in men; U = 127.0, p = 0.244). Similarly, no significant sex-related differences were observed in any of the analyzed laboratory parameters within the odontogenic abscess group (all p > 0.05).

A multivariable logistic regression analysis was performed with abscess origin (nonodontogenic vs. odontogenic) as the dependent variable and CRP, PCT, age, and sex as independent variables. The overall model was statistically significant (p < 0.001). After adjustment for age, sex, and the other inflammatory marker, higher PCT concentrations were independently associated with nonodontogenic abscess origin (adjusted OR = 2.06, 95% CI: 1.23-3.45, p = 0.006), whereas higher CRP concentrations were associated with lower odds of nonodontogenic origin (adjusted OR = 0.981, 95% CI: 0.969-0.992, p = 0.001). Age (p = 0.054) and sex (p = 0.139) were not independently associated with abscess origin.

Spearman’s rank correlation analysis showed significant positive correlations between WBC and CRP in both study groups (nonodontogenic abscesses: ρ = 0.546, p = 0.002; odontogenic abscesses: ρ = 0.585, p < 0.001). In the nonodontogenic group, MPV was negatively correlated with PLT (ρ = -0.466, p = 0.010), while PLT was positively correlated with WBC (ρ = 0.442, p = 0.014). No significant correlation between CRP and PCT was observed in either group (nonodontogenic abscesses: ρ = 0.179, p = 0.345; odontogenic abscesses: ρ = 0.251, p = 0.079).

In sex-stratified analyses, the positive correlation between CRP and WBC remained significant in men with both nonodontogenic (ρ = 0.705, p < 0.001) and odontogenic abscesses (ρ = 0.654, p < 0.001), as well as in women with odontogenic abscesses (ρ = 0.460, p = 0.031). No significant correlation was observed in women with nonodontogenic abscesses (ρ = 0.345, p = 0.328).

Patients were further stratified according to serum PCT concentrations (<0.5 and ≥0.5 ng/mL). MPV, PLT, and MPR were compared between these subgroups using the Mann-Whitney U test. No statistically significant differences were observed in MPV, PLT, or MPR in either the odontogenic or nonodontogenic abscess group (Table 2).

**Table 2:** Comparison of platelet indices according to serum PCT concentrations in patients with odontogenic and nonodontogenic abscesses Data are presented as median (IQR). Comparisons between patients with PCT values ≥0.5 and <0.5 ng/mL were performed using the Mann-Whitney U test. A two-sided p value of <0.05 was considered statistically significant IQR: interquartile range; PCT: procalcitonin; MPV: mean platelet volume; MPR: mean platelet volume-to-platelet count ratio; PLT: platelet

Group	Parameter	PCT ≥ 0.5 ng/mL, median (IQR)	PCT < 0.5 ng/mL, median (IQR)	U	p value
Odontogenic abscesses (n = 17 vs. n = 33)	MPV, fL	8.70 (7.90-9.30)	8.30 (7.70-9.20)	322.5	0.395
	PLT, ×10⁹/L	265 (202-318)	265 (220-327)	264.0	0.743
	MPR	0.0347 (0.0292-0.0437)	0.0309 (0.0267-0.0365)	310.0	0.553
Nonodontogenic abscesses (n = 13 vs. n = 17)	MPV, fL	8.60 (7.90-9.10)	7.70 (7.60-9.20)	125.0	0.557
	PLT, ×10⁹/L	287 (213-299)	248 (205-336)	119.5	0.722
	MPR	0.0288 (0.0258-0.0390)	0.0318 (0.0235-0.0484)	106.0	0.867

## Discussion

The present study focused on the inflammatory profile of odontogenic and nonodontogenic head and neck abscesses, with a particular emphasis on platelet indices and serum inflammatory biomarkers. The main finding was significantly higher serum CRP concentrations in patients with odontogenic abscesses, whereas PCT, PLT, MPV, and MPR did not differ significantly between the groups. However, in multivariable analysis, higher PCT concentrations were independently associated with nonodontogenic abscess origin, whereas higher CRP concentrations were associated with lower odds of nonodontogenic origin, after adjustment for age and sex. In addition, significant positive correlations between WBC and CRP were observed in both groups, while several associations between platelet indices and inflammatory parameters were identified within the nonodontogenic group.

The different behavior of CRP and PCT is noteworthy. While PCT concentrations tended to be higher in nonodontogenic abscesses, CRP was significantly elevated in odontogenic infections. The multivariable analysis further supported this different pattern, as higher PCT remained independently associated with nonodontogenic abscess origin after adjustment for CRP, age, and sex. These findings may reflect the distinct biological behavior of CRP and PCT during bacterial inflammation. CRP is a sensitive but nonspecific acute-phase protein synthesized by the liver in response to interleukin-6 (IL-6) stimulation and may be markedly elevated even in localized bacterial infections. In contrast, PCT is generally more responsive to systemic bacterial inflammation than CRP [8,9]. The independent association between higher PCT and nonodontogenic abscess origin may therefore reflect differences in the inflammatory response between the two types of infection. As these infections often originate from skin and soft-tissue sources, they may involve deeper fascial spaces or present later in the disease course, potentially contributing to the numerically higher PCT concentrations observed in this group. The higher CRP concentrations observed in odontogenic abscesses may reflect differences in the inflammatory response; however, local inflammatory burden was not directly assessed in the present study. IL-6 is a major stimulus for hepatic CRP synthesis and also plays a role in inflammation-associated thrombopoiesis, providing a potential biological link between the acute-phase response and platelet activity during infection [8,9].

The role of MPV in inflammatory diseases remains controversial. Increased MPV has been reported in several chronic inflammatory and cardiovascular disorders, whereas reduced or unchanged MPV values have been described in acute bacterial infections and sepsis [10,11]. A comprehensive review by Korniluk et al. concluded that MPV may behave differently depending on disease type, inflammatory stage, and methodological factors, emphasizing that contradictory findings among published studies are common [12]. Likewise, Gasparyan et al. described MPV as a marker influenced by both thrombopoiesis and inflammatory activation rather than a universal indicator of inflammation [13]. Therefore, the absence of a significant difference in MPV between the two groups is not unexpected and is consistent with the current understanding that platelet indices should be interpreted within the clinical context rather than as isolated biomarkers.

Another important consideration is the considerable analytical variability associated with MPV measurements. MPV is influenced by preanalytical factors including anticoagulant-induced platelet swelling, time between venipuncture and analysis, storage conditions, and differences among hematology analyzers [14,15]. These methodological issues have been identified as major contributors to the inconsistent findings reported in the literature. In the present study, all blood samples were processed using the same analytical platform under standardized laboratory conditions and within a uniform time interval after blood collection, thereby minimizing preanalytical variability and increasing the reliability of the obtained results.

Correlation analysis demonstrated a significant positive relationship between CRP and WBC in both study groups. This finding was expected because both biomarkers represent established indicators of acute bacterial inflammation and confirms the internal consistency of the present dataset [16]. In contrast, an inverse correlation between MPV and PLT was observed in patients with nonodontogenic abscesses. Similar relationships have previously been described in inflammatory disorders and probably reflect physiological regulation of thrombopoiesis, whereby increased platelet consumption is accompanied by accelerated release of larger and metabolically more active platelets from the bone marrow [11,17]. Nevertheless, the biological significance of this association in localized head and neck infections remains uncertain and warrants further investigation. The observed variability in sex-stratified correlations should be interpreted cautiously; however, sex-related differences in inflammatory responses and platelet biology have been previously described [18,19].

From a clinical perspective, our findings suggest that CRP and PCT may provide complementary information on the inflammatory response in patients with head and neck abscesses [20,21]. However, their discriminatory or predictive utility requires further evaluation in larger independent cohorts. Conversely, routine platelet indices appear to provide limited additional value for differentiating odontogenic from nonodontogenic infections. Therefore, MPV and MPR should be interpreted as complementary rather than primary inflammatory biomarkers in this clinical setting.

Recent studies have also investigated combined inflammatory indices and prediction models for risk stratification in deep neck infections. Systemic inflammatory indices, including neutrophil-to-lymphocyte ratio (NLR), platelet-to-lymphocyte ratio, and systemic immune-inflammation index, have shown potential associations with disease severity and clinical outcomes [22,23], while prediction models integrating clinical, laboratory, and imaging parameters may further support clinical decision-making [24]. Serial assessment of inflammatory biomarkers after surgical treatment may also provide additional information on treatment response, although this was not evaluated in the present study.

The present study was performed using the same prospective cohort that was previously used to investigate the diagnostic value of NLR, platelet-to-white blood cell ratio, and MPV in head and neck abscesses [25]. However, the objectives of the current analysis were substantially different. Whereas the previous study focused primarily on leukocyte-derived inflammatory indices, the present work specifically investigated the relationship between PCT, CRP, platelet indices (PLT, MPV, and MPR), and their potential role in distinguishing odontogenic from nonodontogenic abscesses. In addition, the current study includes correlation analyses examining the relationships between inflammatory biomarkers and platelet indices, which were not explored in the previous study.

Limitations

The findings of this study should be interpreted in light of several limitations. First, the study was conducted at a single center and included a relatively small number of participants, which may have limited the statistical power to detect smaller between-group differences, particularly in subgroup analyses. Second, laboratory parameters were assessed at a single time point before surgical drainage, and their dynamic changes during treatment were not evaluated. Information on the exact interval between symptom onset and blood sampling and on antibiotic treatment prior to hospital admission was not systematically recorded; therefore, their potential influence on inflammatory biomarker concentrations could not be assessed. Third, microbiological characteristics and long-term clinical outcomes were not incorporated into the present analysis. Although the multivariable analysis included age and sex, other potential confounders, including symptom duration, previous antibiotic treatment, comorbidities, and measures of disease severity, could not be fully evaluated; therefore, residual confounding cannot be excluded.

## Conclusions

The present study demonstrates different associations of CRP and PCT with abscess origin in patients with head and neck abscesses. Although only CRP differed significantly between the groups in the unadjusted analysis, multivariable analysis showed that higher PCT was independently associated with nonodontogenic abscess origin, whereas higher CRP was associated with lower odds of nonodontogenic origin. Conventional PLT indices showed limited value in distinguishing abscess origin. Future multicenter studies including serial biomarker measurements and detailed microbiological characterization are warranted to further define the role of these biomarkers in the management of head and neck infections.
